# A Two-Turn Shielded-Loop Magnetic Near-Field PCB Probe for Frequencies up to 3 GHz

**DOI:** 10.3390/s23167308

**Published:** 2023-08-21

**Authors:** Mario Filipašić, Martin Dadić

**Affiliations:** Department of Electrical Engineering Fundamentals and Measurements, Faculty of Electrical Engineering and Computing, University of Zagreb, Unska 3, 10000 Zagreb, Croatia; martin.dadic@fer.hr

**Keywords:** magnetic near-field probe, magnetic field measurement, probe sensitivity, electromagnetic compatibility measurements

## Abstract

This paper proposes a novel design of shielded two-turn near-field probe with focus on high sensitivity and high electric field suppression. A comparison of different two-turn loop topologies and their influence on the probe sensitivity in the frequency range up to 3 GHz is presented. Furthermore, a comparison between a single loop probe and a two-turn probe is given and different topologies of the two-turn probe are analyzed and evaluated. The proposed probes were simulated using Ansys HFSS and manufactured on a standard FR4 substrate four-layer printed circuit board (PCB). A measurement setup for determining probe sensitivity and electric field suppression ratio using an in-house made PCB probe stand, vector network analyzer, microstrip line (MSL) and the manufactured probe is presented. It is shown that using a two-turn probe design it is possible to increase the probe sensitivity while minimizing the influence on the probe spatial resolution. The average sensitivity of the proposed two-turn probe compared to the conventional design is increased by 10.1 dB in the frequency range from 10 MHz up to 1 GHz.

## 1. Introduction

Nowadays, as the reliance on electronic devices increases and their number continues to grow even in the fields such as power conversion and systems, electromagnetic compatibility (EMC) is becoming more important than ever. Electromagnetic compatibility, which refers to the ability of electronic devices to operate properly in an electromagnetic environment without being affected by or affecting other electronic devices, is an important factor in regulating these devices. Furthermore, the complexity, level of integration and operational frequencies of such devices is increasing, which results in an increase of electromagnetic pollution they emit into its surroundings. Due to these trends the requirements for EMC certification of electronic devices also becomes harder to achieve. This is an additional challenge for manufacturers, as it requires more time and resources to ensure that their devices meet those standards. Therefore, it is important to take into account that troubleshooting the design of electronic devices may require the use of a certified laboratory, which is an expensive and time-consuming process. An often used and cost-effective method for EMC analysis and pre-compliance testing is the use of an in-house manufactured probe.

Probes can be used for detection and measurement of radiated emissions, which is one of the most common problems designers face during EMC testing. For pre-compliance testing, it is beneficial that the probe is cheap and easily manufactured. Because of that many magnetic near-field probes are manufactured on printed circuit board (PCB) technology. The magnetic field detection depends on the probes characteristics, and based on this, the most important performance factors for a near-field loop probe can be defined:

- sensitivity,
- frequency range,
- electric field suppression ratio,
- spatial resolution.

It is important to note that factors like sensitivity and spatial resolution are inversely proportional. For a higher probe sensitivity the loop area has to be increased, but a bigger loop area lowers the spatial resolution of the probe, therefore making it harder to localize the source of electromagnetic noise. This trade-off between high spatial resolution and a higher sensitivity (especially in lower frequency ranges) is an important challenge for magnetic field probe designers.

Many studies analyzed PCB magnetic field probes and its characteristics. An often used method to increase the electric field suppression of the probe is by shielding. The probe shielding is realized by using the outer layers of the PCB as an electric field shield [1,2]. The loop shield commonly has a gap [1,2,3,4,5] which prevents the flow of shield current. The gap should be placed at the middle of the loop so the structure of the probe is symmetric. In [1] is a wideband shielded-loop probe presented for measurements in the frequency range of 100 MHz up to 6 GHz. An average sensitivity increase of 5 dB is achieved in the analyzed frequency band. The same design optimized for frequencies up to 3 GHz is shown in [6]. A probe with similar frequency range is presented in [2] where the obtained sensitivity of the probe was compared to commercial probes. High spatial resolution and operating frequency is obtained in [7] where the operational frequency of the probe is up to 30 GHz. Multiple probe designs were evaluated in [3] with the goal of increasing electric field suppression ratio of the developed probes with operating frequencies up to 17.8 GHz. A commonly used method to increase the probe sensitivity is by using the resonance phenomena as shown in [8,9,10,11,12]. Resonant probes incorporate a LC resonant circuit into their design by adding additional capacitance into the loop structure. Although such designs offer higher sensitivity at the resonant frequency, they are limited to a narrow frequency band and are not applicable for wideband near-field probes. The main goal of the design in [1] was to increase the sensitivity to the magnetic field, while keeping a high electric field suppression.The high sensitivity of the probe is achieved by incorporating a G-shaped inductive folded line into the design of the loop structure. This probe also incorporates edge plating into its design to increase the electric field suppression ratio. In [13] a two-turn magnetic field PCB probe is presented. Although this design increases the sensitivity in the frequency range up to 3 GHz, the electric field suppression is lower compared to the conventional one-turn shielded probes.

In this paper we focus on the two-turn high sensitive passive probes with high electric field suppression ratio. A two-turn shielded loop magnetic near-field probe is proposed. An analysis between different probe loop structures is given, with a focus on increasing the probe sensitivity. Special consideration is given to developing a probe with two turns and an evaluation of different two turn probe structures is presented. Such an analysis is currently missing from the state-of-the-art literature, as most of the work is focusing on a single turn structure. Furthermore, most of the work is focused on increasing the sensitivity in the frequency range up to 6 GHz [1,2,9] or to further increase the probe usable frequency [7,14]. This work will focus on increasing the sensitivity of the probe in the frequency range up to 1 and 3 GHz, with an emphasis on methods that minimize the influence on the probe spatial resolution. Two distinct frequency ranges are analyzed: 10 MHz up to 1 GHz and 10 MHz up to 3 GHz. Both frequency regions are interesting from an EMC approach. The region from 10 MHz up to 1 GHz covers the common frequency range of 30 MHz up to 1 GHz specified in CISPR 11 radiated emissions limits. Second frequency region from 10 MHz up to 3 GHz is used in test requirements of IEC61967-3 and is commonly defined for commercial magnetic field probes from Langer-EMV (RF-R probes) and Rohde&Schwarz (HZ-17 probes). Furthermore, the effect of PCB edge plating technique on the probe electric field suppression ratio will be analyzed.

## 2. Materials and Methods

### 2.1. Operating Principle

**Figure 1:**
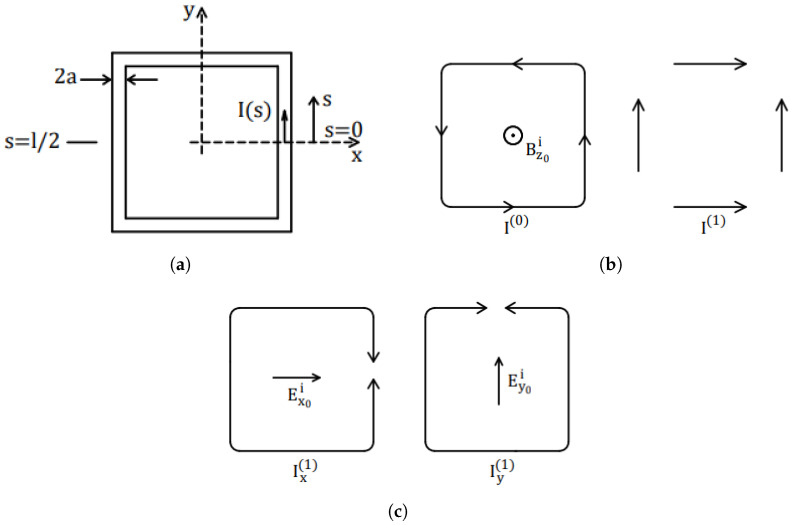
(**a**) Square loop antenna (**b**) Zero- and first-phase-sequence currents induced by magnetic fields (**c**) First-phase-sequence currents $I_{x}^{\left(1\right)}$ and $I_{y}^{\left(1\right)}$ induced by electric field

The operating principle of the loop probe is based on Faraday’s law of electromagnetic induction. In general the probe can be modeled as an electrically small loop antenna with a magnetic and electric-dipole response. The loop probes sustain the circulating current proportional to the magnetic flux density component normal to the plane of the loop, and currents which depend on the electric field tangential to the loop. Figure 1 presents a square loop probe placed in the xy-plane of the coordinate system, where variable *s* denotes the line coordinate along its perimeter. We also assume that the loop is immersed in a linear homogeneous isotropic medium with magnetic and electric properties of vacuum.

Let the perimeter of the loop be equal to *l* and *a* is radius of the conductor. We also assume that *a* is sufficiently small to allow only one-dimensional flow of the current, and time-harmonic incident field, written in complex notation as $e^{j\omega t}=e^{jkct}$. Here $j=\sqrt{-1}$, ω is radian frequency, *t* denotes time, *c* is speed of light, λ is wavelength and k=2π/λ is wave number. We now write the Faraday’s law of induction in phasor notation as

(1) $$-jk\int_{s}cB\;\mathrm{dS}=\oint_{s}E\;\mathrm{ds}.$$

Here magnetic flux density B is the sum of incident component $B^{i}$ and reradiated component $B^{r}$. The total current can be expressed as a sum of zero-phase sequence $I^{0}\left(s\right)$ and first-phase sequence $I^{1}\left(s\right)$:

(2) $$I\left(s\right)=I^{0}\left(s\right)+I^{1}\left(s\right)$$

as depicted in Figure 1b. Incident field $B^{i}$ and radiated fields $E^{r}$ and $B^{r}$ can be expressed using components $I^{0}\left(s\right)$ and $I^{1}\left(s\right)$. The zero-phase sequence current is associated with $B^{i}$ as [15]:

(3) $$-jk{\;\int \int \;}_{s}cB^{i}\;\mathrm{dS}=\oint_{s}I^{\left(0\right)}\left(s\right)Z^{i}\;ds+\frac{j\omega \mu }{4\pi }\oint \oint I^{\left(0\right)}\left(s\right)\frac{e^{-jkR}}{R}\;\mathrm{dsds}$$

Here $Z^{i}$ is the internal impedance per unit length, μ is magnetic permeability, while *R* is the distance from source point to field point. For electrically small loops, we may write, using first-order approximation [15]

(4) $${\;\int \int \;}_{s}cB^{i}\;\mathrm{dS}=cB_{z_{0}}^{i}S$$

where $B_{z_{0}}^{i}$ is the normal component of magnetic flux density in the center, *S* is area of the loop, and $i\left(s\right)=I_{0}$, resulting in magnetic effective length as

(5) $$h_{b}=-jkS$$

The electric field response is dependent on the first-phase-sequence current $I^{\left(1\right)}$, which can be further broken up in two parts, $I_{x}^{\left(1\right)}$ and $I_{y}^{\left(1\right)}$ as in Figure 1c. They are dependent on the respective electric field components $E_{x_{0}}^{i}$ and $E_{y_{0}}^{i}$. For the loads at s=0, $I_{x}^{i}$ can be ignored. As well, $I_{y}^{\left(1\right)}$ vanishes at s=±l/4, allowing the representation of the loop as an array of two U-shaped antennas, which electric effective length can be determined [15] using Rayleigh-Carson reciprocal theorem and a short dipole antenna in its far-zone field. We further define unloaded magnetic sensitivity

(6) $$K_{B}=\frac{Y_{0}h_{b}}{\lambda }$$

and unloaded electric sensitivity

(7) $$K_{E}=\frac{Y_{I}h_{eI}}{\lambda }$$

according to [15], both expressed in $\Omega^{-1}$. Here $h_{eI}$ is electric effective length of each U-shaped “dipole”. Also, $Y_{0}$ is low-frequency input admittance of the loop (with I(s)=const.):

(8) $$Y_{0}={\left[\oint Z^{i}ds+\frac{j\omega \mu }{4\pi }\oint \oint \frac{e^{-jkR}}{R}\;\mathrm{dsds}\right]}^{-1}$$

and $Y_{I}$ is the input admittance at the centers of half-dipoles. A loaded loop at s=0 can be described using loaded sensitivity constants

(9) $$K_{B}^{\left(1\right)}=\frac{Y_{L}}{Y_{L}+Y}K_{B}$$

(10) $$K_{E}^{\left(1\right)}=\frac{Y_{L}}{Y_{L}+Y}K_{E}.$$

As a measure of the electric field influence, alongside with [15], we define system error ratio

(11) $$e=\frac{K_{E}^{\left(1\right)}}{K_{B}^{\left(1\right)}}$$

as the ratio of the output current caused by unit parallel electric field $E_{y_{o}}^{i}=1$ V/m, and the output current caused by weighted unit magnetic flux density $cB_{z_{o}}^{i}=1$ V/m. Here, the magnetic flux density is multiplied by speed of light *c* to express both fields in the same units V/m.

For a square loop with side w≤0.03λ, we define the loaded sensitivity constants according to [15]

(12) $$K_{B}^{\left(1\right)}=\frac{Y_{L}}{Y_{L}+Y^{\left(0\right)}}\frac{-\pi w}{\lambda \xi (\Omega -4.32)}$$

(13) $$K_{E}^{\left(1\right)}=\frac{Y_{L}}{Y_{L}+Y^{\left(0\right)}}\frac{j3\pi^{2}w^{2}}{\lambda^{2}\xi \left(\Omega -3.17\right)}$$

where zero- and first-phase-sequence components of the input admittance are defined as

(14) $$Y^{\left(0\right)}=-j\lambda {\left[2w\xi (\Omega -4.32)\right]}^{-1}$$

(15) $$Y^{\left(1\right)}=j2\pi^{2}w{\left[\lambda \xi (\Omega -3.17)\right]}^{-1}.$$

Here ξ is characteristic impedance of the medium (120π for free space), *w* is side of square loop and wire thickness parameter Ω=2ln(4w/a). System error ratio for a square loop is defined as

(16) $$e=-j3\pi \frac{w}{\lambda }\frac{\Omega -4.32}{\Omega -3.17}.$$

We see that system error ratio tends to zero as $\frac{w}{\lambda }\to 0$. For small square loops, magnetic length is [15]:

(17) $$h_{b}=-jkw^{2}.$$

Consequently, corresponding open-circuit voltage is

(18) $$V_{H}=h_{b}cB_{z}^{i}=-j\omega \frac{S}{c}B_{z}^{i}=-j\omega S\mu H_{z}^{i}$$

where $H_{z}^{i}$ is normal component of the incident magnetic field vector. As well, the effective electric length is [15]

(19) $$h_{eI}=\frac{\sin\frac{1}{2}kw-\cos kw}{k\sin\frac{1}{2}kw}$$

and the input admittance is

(20) $$Y_{I}=j2\pi \tan\frac{kw}{\xi (\Omega -3.17)}.$$

Consequently, corresponding open-circuit voltage is

(21) $$V_{E}=h_{eI}E_{y_{0}}^{i}.$$

Equations (19) and (20) are applicable for w≤0.1λ. Using approximation tan(kw)=kw for small arguments kw, from reduced form $K_{E}^{\left(1\right)}$ defined in (13) and from (7) and (10) we arrive at further approximation

(22) $$h_{eI}=\frac{3}{4}w$$

valid for w≤0.03λ. In [16], a similar analysis is presented, suitable for arbitrary shapes of receiving coils.

Calibration of near-field probes is based on the measurements using TEM cells [17], Helmholtz coils [18,19] and transmission line on a planar substrate [1,6,13,17]. Microstrip line (MSL) calibration is also required by standards IEC TS 61967-3 and IEC 61967-6. However, evaluation of the produced field is not straightforward [17], while most papers on NFP calibration use 3D electromagnetic simulation [17,20,21,22,23,24,25]. In the sequel, we will also perform numerical FEM simulation of the calibration setup. Note that [17] provides several closed-form expressions of the near E- and H-fields for a MSL calibration setup of finite length. As well, [26] and IEC 61067-6 gives simple expressions for MSL setup based on the infinitely long thin wire approximation.

**Figure 2:**
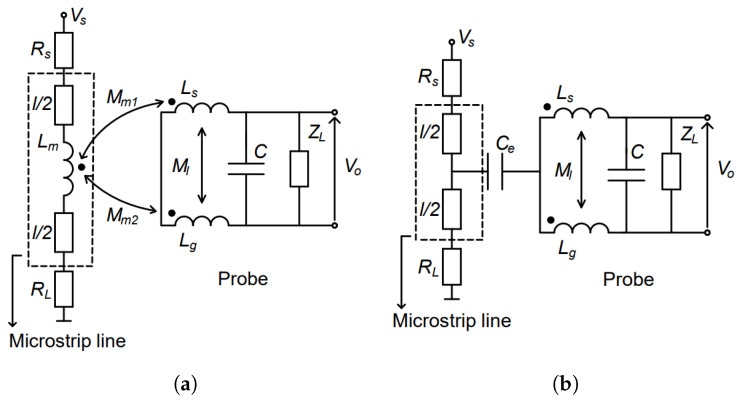
(**a**) Equivalent circuit for magnetic coupling mechanism from MSL (microstrip line) to loop antenna (**b**) Equivalent circuit for electric field coupling mechanism from MSL to loop antenna

Figure 2 illustrates magnetic and electric field coupling mechanism. Figure 2a presents the equivalent circuit for the magnetic field coupling of the measurement system, consisting of conventional shielded loop probe and MSL according to [1]. $R_{s}$ is source resistance of the network analyzer, while $R_{L}$ denotes matched load resistance of MSL, i.e., $R_{L}=50$Ω. $L_{s}$ is the self-inductance of the receiving loop, placed in the inner layer. $L_{g}$ is self-inductance associated with the magnetic flux in the ground loops, forming between feeding line and ground planes which serve as shield and return conductors. $M_{l}$ is mutual inductance between ground loops and the receiving loop. Finally, *C* includes inner capacitance of the loop and capacitance between ground planes and inner line conductor (incorporating receiving loop and feeding line). $M_{m_{1}}$ presents mutual inductance between MSL and receiving loop, and $M_{m_{2}}$ denotes mutual inductance between MSL and ground loops. Inductances $L_{m}$, $M_{m_{1}}$ and $M_{m_{2}}$ are used to describe magnetic field coupling mechanism. $Z_{L}$ is the load impedance of the probe, i.e., the load imepdance of the receiving port of the network analyzer. Figure 2b presents the equivalent circuit for the electric field coupling [3]. Here, $R_{s}$ is again source resistance, $R_{L}$ is load resistance, $Z_{L}$ is impedance of the receiving port. $C_{e}$ is coupling capacitance between MSL and the probe (representing coupling mechanism for the electric field). Finally, *C* is overall capacitance of the probe.

In general, the probe can be divided into 2 main parts: the sensory part and the transmission part. The sensory part is composed of the inductive loop and the shield aperture, which is a cut-out region in the ground plane on both top and bottom layers. To maintain a symmetric structure the loop shield is split at the middle of the loop. The symmetric structure enables the cancellation of common-mode currents induced by the external electric field on the external surface of the shield [27]. The presence of unbalanced currents in the shield would generate a secondary magnetic field that, in turn, induces an additional current in the inductive loop. This secondary magnetic field adds an additional error to the measurement of the primary *H*-field. In this paper five different probes are designed with the same inner loop dimensions and total probe dimensions. With such starting conditions the effect of the loop structure and change in its self-inductance and self-capacitance can be further analyzed. All of the designed probes use a rectangular planar loop and are manufactured on a standard FR4 substrate ($\epsilon_{r}=4.4$) with 4 layers. The probe structure is based on a stripline circuit, with the probe loop on the inner layers and ground plane on outer layers.

### 2.2. Benchmark Probe

**Figure 3:**
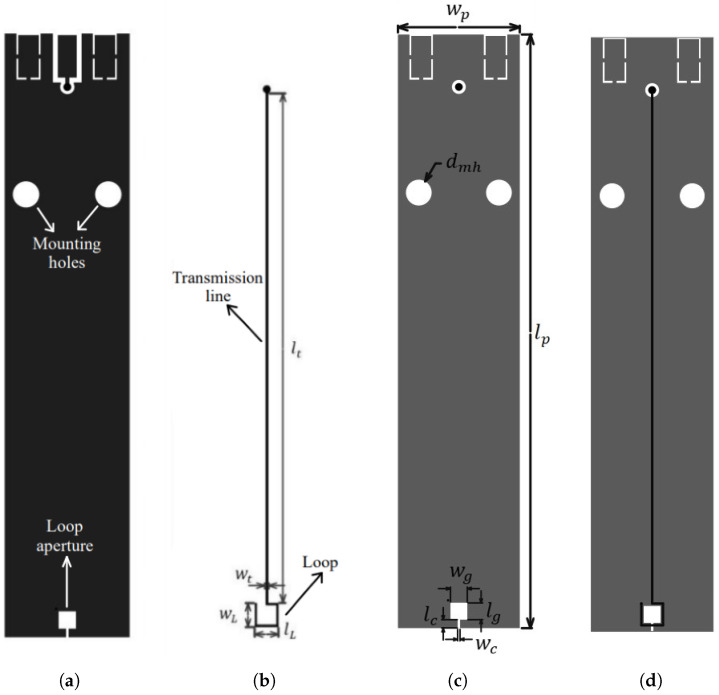
Depiction of the conventional probe dimensions. The figure shows different probe layers: (**a**) top layer (L1), (**b**) inner layer 1 (L2), (**c**) bottom layer (L4) and (**d**) inner layer 1 (L2) and bottom layer (L4).

For the analysis, a conventional probe with a single turn is designed which will serve as a benchmark for other probes. The probe outer dimensions are 15.2 mm width and 70 mm length with a 3 mm × 3 mm loop area. The structure and general shape of the conventional probe is shown on Figure 3 where its dimensions are marked and further described in Table 1.

During the design stage of the conventional probe, emphasis is given to keeping its dimensions compact and taking standard manufacturer capabilities into consideration. The initial design was optimized to increase the probe sensitivity in the frequency range from 10 MHz to 3 GHz. The probe initial loop area was selected according to similar probes in the literature [1,2,12] for the frequency range of interest. The probe sensitivity dependence on loop area is simulated using a parametric sweep to find the best fitting values for increased sensitivity in the analyzed frequency range. The loop dimensions are given as $w_{L}$ × $l_{L}$, and the parametric sweep consisted of loop starting values 1 mm × 1 mm up to 3 mm × 3 mm with a step of 0.5 mm. The step was selected due to manufacturing tolerances. The highest loop sensitivity is achieved according to Equation (18) for the highest loop area. Although higher loop dimensions would achieve higher sensitivity, the loop dimensions of 3 mm × 3 mm are selected, as these are considered a good balance between sensitivity and spatial resolution. For the obtained loop area the probe outer dimensions were further optimized so that resonances in the probe structure were minimized. Resonances in the probe arise due to impedance mismatch and the general probe structure [28]. By changing the length of the probe, the transmission line length was changed accordingly. This way the mismatch between the probe input impedance and the measurement apparatus impedance can be minimized. The optimization process included a parametric sweep for obtaining initial results. According to the obtained values the HFSS built-in PSO algorithm was used to determine the best fitting values for probe width and length. The goal of the optimization was to increase the sensitivity in the frequency range from 10 MHz to 3 GHz, while decreasing the resonances in the probe operating frequency range. The optimization variables were probe width $w_{p}$, probe length $L_{p}$, line width $w_{t}$, aperture width $w_{g}$ and aperture length $L_{g}$. The value of the aperture width and length is kept the same to maintain a rectangular shape of the aperture. The range of the optimization variables is shown in Table 2.

In the PSO implementation, a maximum of 100 iterations are allowed for attaining a cost function value of 0.1. The goal of the optimization is achieving a $S_{21}\geq -35$ dB at frequencies from 800–3000 MHz. The final obtained dimensions of the probe are as shown in Table 1.

The layer stack-up of the conventional probe is shown in Table 3 and the material and thickness of each layer is given. During the design of the proposed probes the dielectric thickness $h_{d_{1}}$ and $h_{d_{3}}$ are lowered to 0.2 mm. With this approach the coupling between the ground plane and the probe loop is increased, resulting in an increase of the induced current in the loop. While this approach enhances the sensitivity of the probe, it simultaneously raises the parasitic capacitance between the ground plane and the loop. This reduces the resonant frequency of the probe, but from a perspective of increasing the sensitivity in the lower frequency range it is a valid approach.

### 2.3. Two-Turn Probe

**Figure 4:**
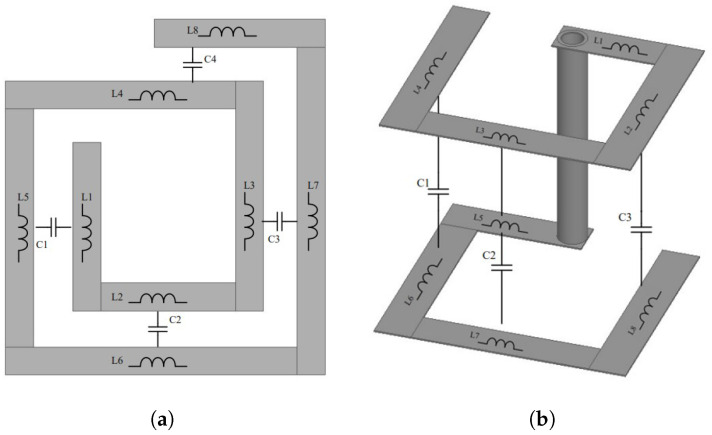
Partial inductance and parasitic capacitance of the (**a**) planar loop and (**b**) basic two-layer loop topology.

The primary focus during the initial design stage is to enhance sensitivity towards magnetic field reception, while simultaneously maintaining the existing spatial resolution. Therefore, two distinct approaches are used in this paper to design a two-turn probe: one involves placing the second turn on the same layer as the first turn effectively making a planar loop, while the second method places each turn on a separate inner layer. This way the inductance of the loop is increased and additional parasitic capacitance is introduced, therefore lowering the resonant frequency of the probe. In the current literature there is a lack of two-turn probes developed on separate layers of the PCB stack-up. To the best of the authors knowledge, there is a two-turn probe design for an active magnetic field probe proposed in [13]. Although the design in [13] increases the sensitivity of the probe in the frequency range up to 3 GHz, the electric field suppression ratio is lower than the standard one-turn shielded probe design. The inductance and parasitic capacitance of basic loop structures are shown in Figure 4. Additional shielding is achieved by adding edge plating to the probes. This is an alternative to the via fence technique [1,3,29] and it is shown that such shielding is an effective way for suppressing resonances in the probe operational frequency range. Edge plating is a manufacturing process where the PCB side edges are plated with copper from the top to the bottom layer. Edge plating is an often-used method [30,31] in radiofrequency (RF) circuit design to increase the shielding and reduce electromagnetic interference (EMI) from PCB circuits. Both studies show that edge plating is a more effective method for shielding boards than stitching vias. The downside of edge plating is the increased cost of manufacturing compared to via stitching. In [1] it is also efficiently implemented in a shielded-loop magnetic near-field probe.

It can be seen that to increase the resonant frequency of the probe it is important to lower the parasitic capacitance and vice versa. In the planar loop topology this is achieved by increasing the distance between the loops. This can be done by increasing the outer loop diameter which would result in deteriorated spatial resolution of the probe. Another method is to decrease the inner loop diameter which as a result would decrease the probe effective area and therefore decrease the probe sensitivity. This effect is further enhanced because the shield cut-out is usually smaller than the inner loop diameter. The topology with loops on separate layers doesn’t have this limitations as the parasitic capacitance is mainly determined by substrate height.

The partial inductances and capacitances of loops in Figure 4 are depicted according to [32], for wireless power transfer (WPT) coils, where an analysis of inductance and parasitic capacitance is carried out with similar space configuration of coils.

## 3. FEM Simulations

**Figure 5:**
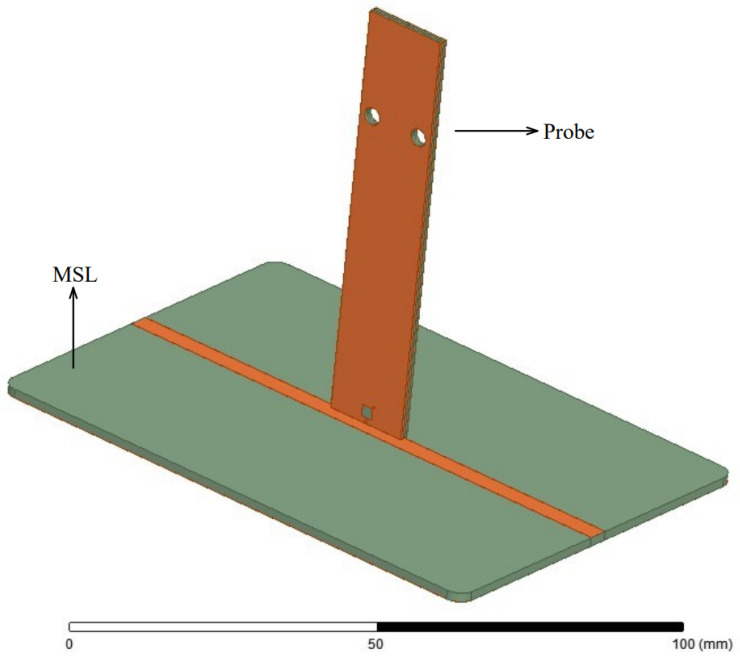
3D simulation model of the probe and MSL developed in Ansys HFSS used for determining S-parameters of the probe.

The designed probes were simulated using Finite Element Method (FEM) simulation models, developed in the electromagnetic full-wave solver software Ansys HFSS [33]. A 3D simulation model for simulating the probe sensitivity is developed using a 50 Ω MSL, shown in Figure 5, which is an often used method to verify the probe sensitivity [1,2,3,8,9,10,11,14]. Three ports from which the *S*-parameters will be analyzed are defined on the simulation model: Port 1 for MSL input, Port 2 for probe output, Port 3 for MSL termination.

**Figure 6:**
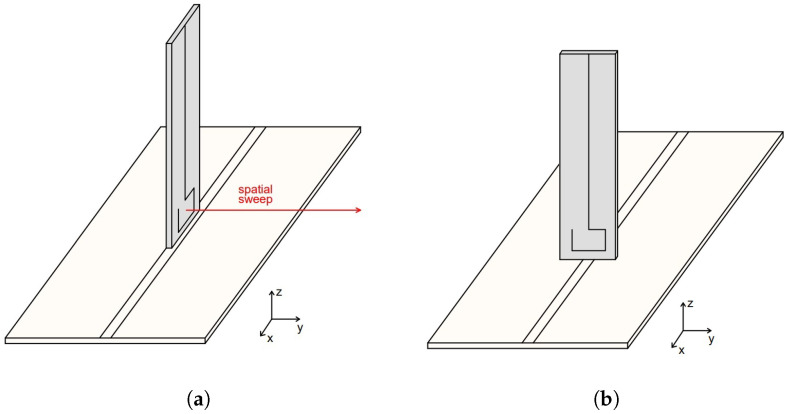
Probe and MSL configuration for (**a**) measuring maximum magnetic field and (**b**) measuring minimum magnetic field. In (**a**) the spatial sweep direction for the spatial resolution measurement is shown.

In the simulations the probe tip is placed 1 mm above the middle of the MSL. By using the aforementioned ports, input of the MSL as port 1 and probe output as port 2, the sensitivity of the probe can be expressed in terms of $S_{21}$ parameter. The MSL in Figure 6a is placed horizontal in the xy-plane and the probe is placed over the center of the MSL so that the plane of the probe loop is parallel to the xz-plane. In this configuration the probe picks up the maximum magnetic field and the electric field is coupled from the MSL to the probe. When the probe is rotated by 90° around the z-axis, according to Figure 6b, it picks up the minimum magnetic field and again the electric field is coupled to the probe. Because of this effect the electric field suppression ratio of the probe ($SR_{H/E}$) can be evaluated as the difference between the $|S_{21}|$ parameter in each case.

**Figure 7:**
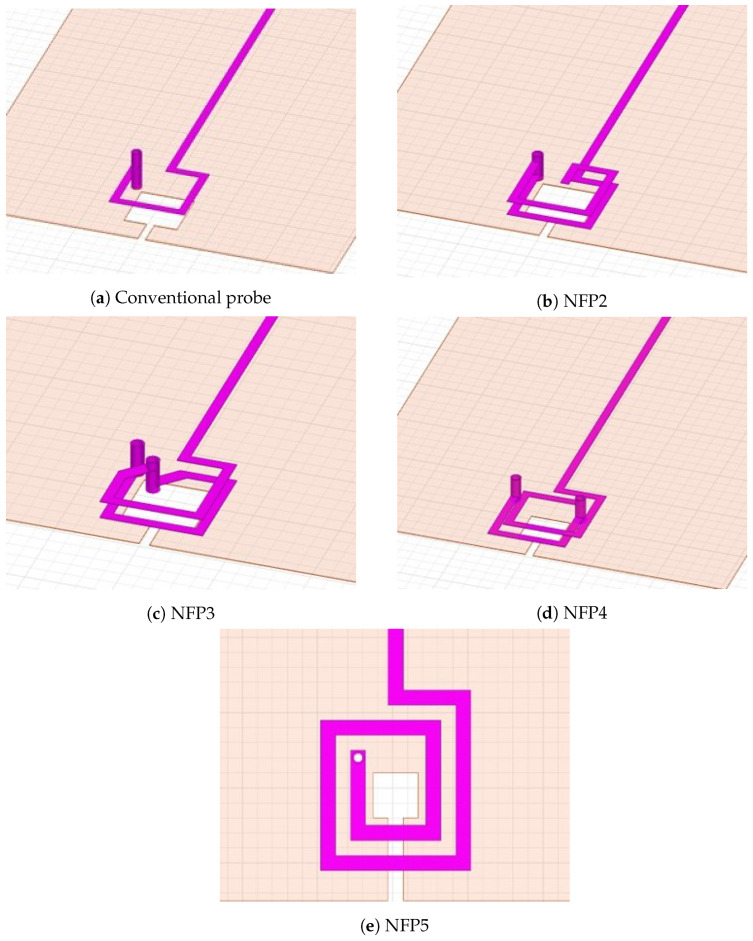
3D model of the probe loop structure for each of the designed probes in Ansys HFSS. In (**a**) the conventional probe loop is shown, (**b**) shows probe with a folded line, (**c**,**d**) are 2 layer two turn probes, and (**e**) is a two-turn planar loop probe.

The Figure 7 shows five designed loop routing topologies which will be further analyzed. The probes are called near-field probe (NFP) and are marked as NFP1 up to NFP5. The conventional probe (NFP1), depicted in Figure 7a is the benchmark probe for all new designs. This design is often used for comparison because of the simplicity, good sensitivity and electric field suppression ratio and therefore is considered a conventional design [1,3,8,13]. During the design process the goal was to keep the same loop dimensions (3 mm × 3 mm) in the new designs. This way the loop structure effect on the probe sensitivity can be analyzed without major changes in the probe spatial resolution. The probe structure NFP2 in Figure 7b follows the design in [1], which implemented the folded line technique to increase probe self-inductance. NFP2 is further optimized using the Ansys HFSS built-in PSO algorithm and analyzed in [6]. The goal of the optimization is to increase the sensitivity in the frequency range up to 3 GHz, while keeping the loop outer dimensions 3 mm × 3 mm. The increase in self-inductance and self-capacitance due to the folded line adds a self-resonance in the frequency range between 2 and 3 GHz, which increases the probe sensitivity. The highest usable frequency of the probe is generally defined as a little higher than its self-resonance frequency.

The NFP3 in Figure 7c shows a novel two-turn loop structure where the loops are parallel to each other. The loop area of the designed probe is 3 mm × 3 mm. In the NFP3 design the focus is on a high sensitivity passive probe with high electric field suppression ratio. The downside of NFP3 design is the interconnecting copper track between the loop layers, which is made with a diagonal segment across the loop aperture. This way the outer shield of the probe is kept symmetrical, but the diagonal interconnection loop part is not shielded. The NFP4 shown in Figure 7d, is a novel design developed to avoid the non-shielded diagonal interconnection copper track. The probe has turns on separate layers which are shifted for easier interconnection with a via between them. It is important to note that although the unshielded track is avoided here, the layer 2 and 3 interconnection via breaks up the symmetrical structure of the outer shield. The loop area of each loop is 3 mm × 3 mm, while the total area of the sensory part is 3.6 mm × 3 mm. An analysis of electric field suppression ratio between all designs will be presented in the sequel (Section 4).

Furthermore, a novel planar two-turn probe NFP5 is designed as shown in Figure 7e. As most of the research analyzes single-turn shielded PCB probes, there is a lack of investigation on shielded probes based on a single-layer planar coil design. The NFP5 is a two turn planar coil developed on a four-layer PCB with the planar coil on inner layer 2. The probe loop dimensions are 3 mm × 3 mm for the inner loop area and 5 mm × 5 mm for the outer loop area. During the design stage it is decided that the inner loop area of 3 mm × 3 mm will be identical to the other designed probes, while the outer loop area is 5 mm × 5 mm. This allows for the design to have the same loop aperture as other probes. During the design process of the probe the inner loop area is considered a fixed parameter and only spacing between the turns is changed. The goal of the design stage is to achieve the highest sensitivity in the frequency range up to 1 GHz. The obtained spacing between turns is 0.5 mm.

It is noteworthy to discuss further increase in the sensitivity of the probe by incorporating multiple turns. This can be achieved by using a PCB stack-up with more layers (6 or 8), but this would also increase the manufacturing cost of the probe. The second approach is to add more planar turns. Both approaches of increasing the number of turns have a downside of increasing the probe self-capacitance and self-inductance which considerably lowers the probe usable frequency range. A similar approach is described in [19] where a seven-turn planar unshielded magnetic field probe on a two-layer stack-up was designed with a resonant frequency of 77 MHz. This probe could be further shielded to increase the electric field suppression ratio, but due to the higher number of turns the loop aperture would be small compared to the total probe dimensions, which would diminish the sensitivity of the probe.

## 4. Results and Discussion

### 4.1. Experimental Setup

**Figure 8:**
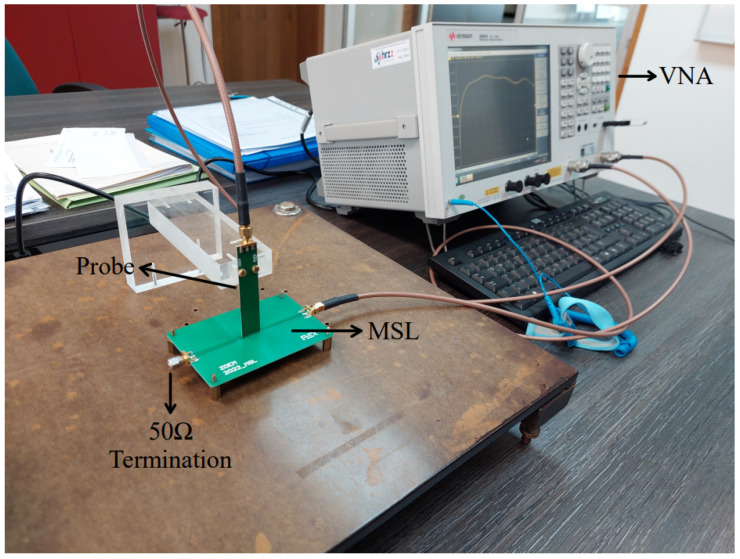
Measurement setup for evaluating probe sensitivity consisting of the probe, MSL and VNA (vector network analyzer).

The experimental setup for verifying the design and performance of the manufactured probes aligns with the simulation setup, as depicted in Figure 8.

**Figure 9:**
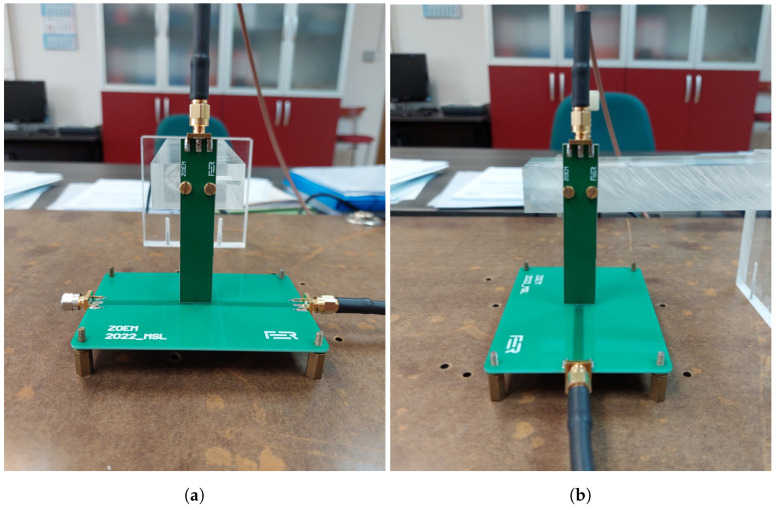
Measurement setup for evaluating probe (**a**) sensitivity and (**b**) electric field suppression ratio. The figures show the probe fixation with screws and MSL fixation with standoffs in both measurement setups.

**Figure 10:**
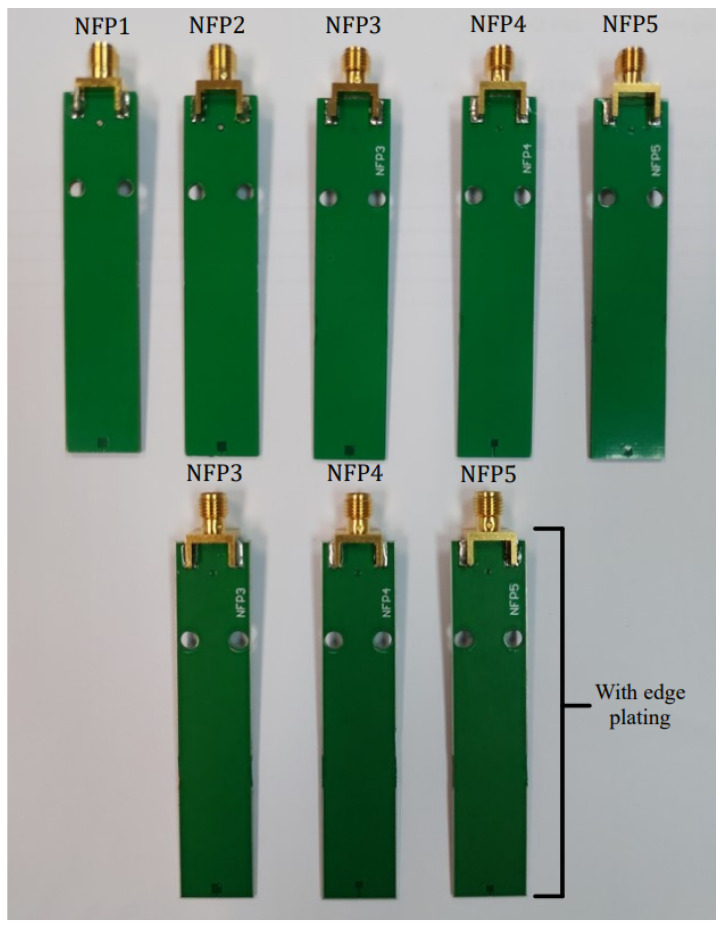
Manufactured near-field probes, top row are probes without edge plating and bottom row are edge plated probes.

A 50 Ω MSL has been designed and manufactured on FR408HR substrate with a 50 Ω SMA termination rated up to 4 GHz. The MSL consists of two layers: top layer where the microstrip line is placed and the bottom ground layer. The dimensions of the MSL are 100 mm length, 70 mm width, 1.55 mm substrate thickness and the line conductor of 3.5 mm width. The vector network analyzer (VNA) used for measurements is Keysight E5061B (option 3L5) with a measurement uncertainty of below 1.0 dB for the measurement range of 10 MHz up to 3 GHz. The VNA is calibrated utilizing the Keysight 85033E calibration kit. An in-house manufactured PCB probe stand is designed with emphasis on fixation of the probe to minimize the measurement errors due to probe displacement. This is an cost-effective alternative to the commonly used automatic scanning systems [7,10] where the probe is attached to a robot arm. Each probe is manufactured with 2 holes for screw fixation to the stand as shown in Figure 9. The measured distance between the probe tip and MSL is 1 mm. The manufactured probes NFP1 (conventional), NFP2, NFP3, NFP4 and NFP5 without edge plating and NFP3, NFP4 and NFP5 with edge plating are shown on Figure 10.

First measurement step is determining the probe $S_{21}$ parameter for sensitivity to the magnetic field radiated from the MSL. The probe and MSL in the maximum magnetic field pickup setup are shown in Figure 9a. Next the MSL is rotated for 90° around the z-axis as in Figure 9b and the measurement of the $S_{21}$ parameter is repeated. After obtaining both $S_{21}$ parameters in dB, the electric field suppression ratio $SR_{H/E}$ can be determined according to

(23) $$SR_{H/E}=S_{21,H}-S_{21,E}$$

where $S_{21,H}$ is the parameter obtained for maximum magnetic field setup and $S_{21,E}$ the parameter for minimum magnetic field setup. During both measurements the probe tip is kept at a fixed distance of 1 mm above the center of the MSL. The PCB probe stand is designed so the MSL can be easily rotated and fixated with screws, allowing for good repeatability of the measurements and probe position above the center of the MSL. The stand incorporates two holes for probe mounting in both measurement arrangements. This way either the probe or the MSL can be rotated during the measurements.

### 4.2. Sensitivity Measurements

**Figure 11:**
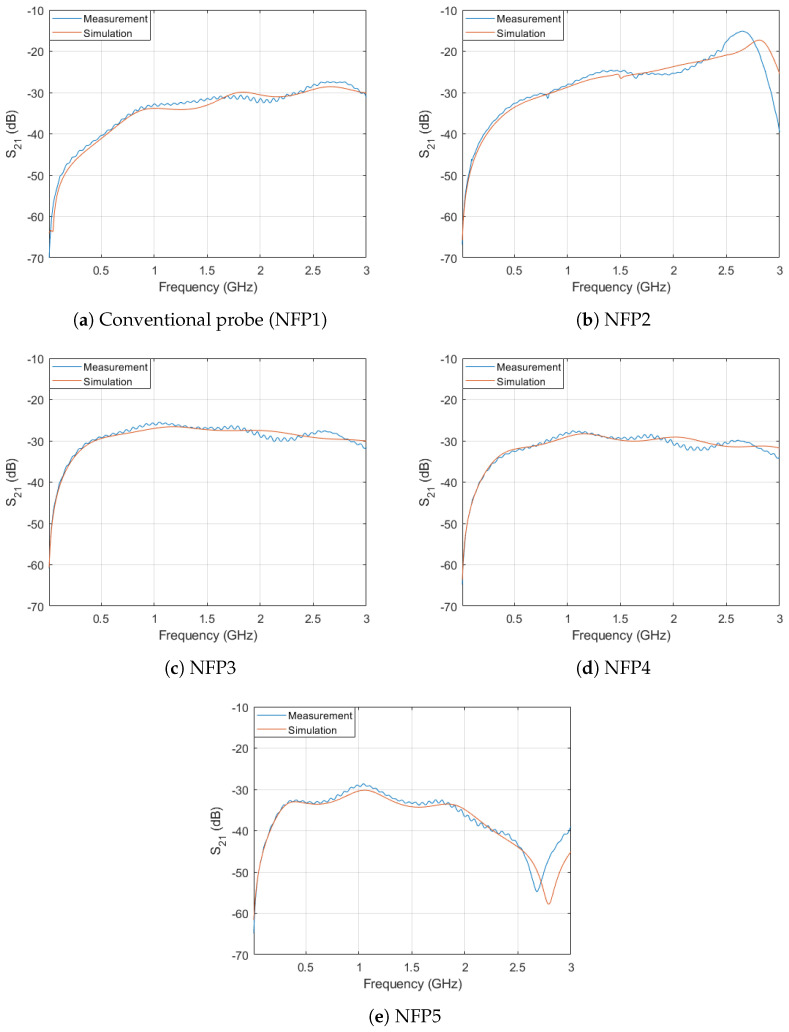
Comparison of simulated and measured sensitivity of the probes.

The manufactured probes sensitivity obtained by FEM simulation is validated against measurements and the comparison is shown in Figure 11. The results show a good correspondence between the measured values and the simulated values up to the frequency ranges of 2 GHz. Above 2 GHz the results have a slight mismatch which is due to manufacturer production tolerances and reflections on the SMA connectors which are not included in the 3D FEM model.

Comparing the sensitivity measurement results in Figure 11 and Table 4 it can be seen that NFP3 has the highest magnetic field sensitivity in the lower frequency region (below 1.5 GHz). Probe NFP2 offers the highest sensitivity in the frequency range from 1.5 GHz up to the resonant frequency at 2.7 GHz. The probe NFP4 has an increase in sensitivity in the frequency range up to 2 GHz when compared to the conventional probe. When compared with the NFP3 probe design the sensitivity of the NFP4 is lower in the whole observed frequency range. The planar two-turn NFP5 probe design mainly increases the probe sensitivity in the frequency range up to 1 GHz and after 2 GHz the probe sensitivity substantially drops. The probe shows a resonance phenomena in the frequency range between 2.5 and 3 GHz.

**Figure 12:**
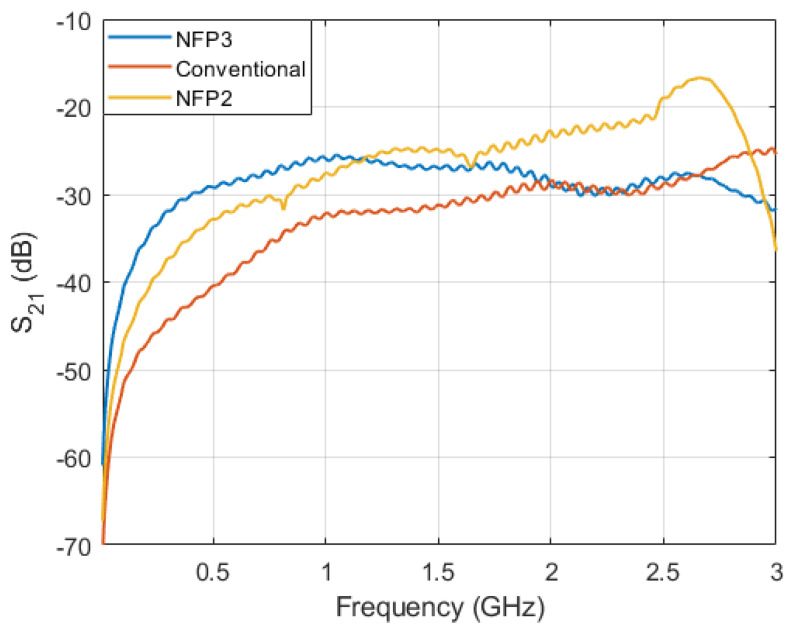
Comparison of the NFP1 (conventional), NFP2 and NFP3 probe measured sensitivity.

Further analysis is made between probe NFP2 and NFP3 as they are the best performing probes of 5 analyzed topologies. These two probes have the highest sensitivity in the analyzed lower and higher frequency ranges respectively. Both of the probes are also compared to the benchmark probe. The comparison between their sensitivity is shown in Figure 12. The NFP3 probe sensitivity in the frequency range from 10 MHz up to 1 GHz is increased by an average of 10.11 dB compared to the conventional probe and 4.24 dB compared to the NFP2 probe. The NFP3 probe shows an almost flat response from 300 MHz up to 3 GHz without any significant resonances. The NFP2 probe which uses a folded line [1] to increase the probe self-inductance has a smooth response up to 2 GHz, after which the sensitivity increases considerably due to the resonance at 2.7 GHz and decreases above that frequency. When compared with resonant probes in [8,9,10,11,12] which have a high sensitivity in a narrow band around the resonant frequency, this probe also offers a higher sensitivity due to resonance but is suitable for broadband measurements up to the resonant frequency. It is important to note that because the NFP3 probe maintains the same outer loop dimensions as NFP2 and the conventional probe, the spatial resolution is not significantly affected by this design technique. The outer loop dimensions of the NFP2 and conventional probe must be increased (which would result in lowering their spatial resolution) to achieve a comparable sensitivity to the NFP3 probe in the lower frequency ranges.

Sensitivity at frequencies of interest for each of the manufactured probes is shown in Table 4 and the measured and simulated results are compared. The addition of the edge plating has only a small influence on probe sensitivity and the main contribution is in decreasing and smoothing out the resonant peaks of the probe. The main effect of the edge plating on electric field suppression ratio is discussed in next subsection. Figure 12 graphically depicts measured sensitivity of NFP1, NFP2 and NFP3.

It can be noted that for frequencies above 2.5 GHz the conventional probe design shows a higher sensitivity than the proposed probe NFP3. The conventional design has a simulated sensitivity of −29.3, −30.4, −31.4 and −27.4 dB at frequencies 2.5, 4, 5 and 6 GHz, whereas the NFP3 has a simulated sensitivity of −29.8, −36.8, −37.9 and −29.7 dB at those frequencies. The key factor for the increased sensitivity of the NFP3 probe in the frequency range up to 2.5 GHz is due to the increased inductance of the probe by incorporating an additional turn. While the dimensions of the loop width and aperture area of the conventional design and NFP3 are kept identical, the probe NFP3 shows a better sensitivity at lower frequencies.

**Figure 13:**
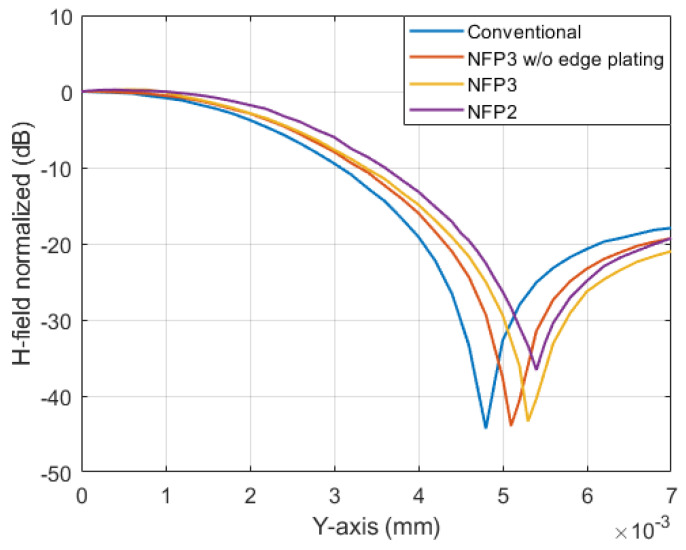
Comparison of the conventional (NFP1), NFP2 and NFP3 (with and without edge plating) probes spatial resolution at frequency of 1 GHz.

The spatial resolution of the conventional, NFP2 and NFP3 probe is compared through simulation. A comparison between NFP3 with and without edge plating is given too. The spatial resolution is not measured because the measurement setup is not appropriate for consistent small positional changes. The probe is placed in the center of the MSL and moved along the y-axis as shown in Figure 6a and Figure 9a. The spatial sweep step of the simulation is 100 µm. This way the normalized magnetic field distribution over the MSL is obtained and shown in Figure 13. The normalized magnetic field response is simulated at 1 GHz for each of the analyzed probes. As described in [3] the spatial resolution is defined as the point of 6 dB level reduction from the peak magnetic field point in the center of the MSL. For the conventional probe the obtained spatial resolution is 2.5 mm. The NFP3 probe with and without edge plating has a simulated spatial resolution of 2.7 mm, although the NFP3 with edge plating shows a slight deterioration of the spatial resolution. The NFP2 probe has the lowest spatial resolution at 3 mm. Further research can be carried out on the effect of edge plating on probe spatial resolution, as the simulation results show a slight change. In addition, a comparison between edge plating and via stitching influence on spatial resolution can be done in the future.

Direct comparison between parameters of the proposed and the two-turn probe in [13] is a challenging task.The main problem is the sensitivity and spatial resolution measurement dependence on the probe position above the MSL. While the measurement setup in this paper has the probe tip 1 mm above the MSL, the distance in [13] is 0.5 mm affecting the measured probe sensitivity. The probe distance from MSL also affect the measured spatial resolution of the probe as discussed in [3], which shows that for a higher distance the spatial resolution is deteriorated. In this paper we also propose passive probes, while [13] design is based on an active probe. The simulation results of the two-turn probe structure in [13] show a sensitivity of around −30 dB at 3 GHz and −31 dB at 3 GHz. In [1] the probe tip is placed 1 mm above MSL, which allows for better comparison of results with our probe. Their calculated sensitivity is −39.5 dB at 1 GHz and −32.3 dB at 3 GHz, while the proposed probe NFP3 has a simulated sensitivity of −26.5 and −31.6 dB at frequencies 1 and 3 GHz respectively.

### 4.3. Edge Plating Influence on Electric Field Suppression

**Figure 14:**
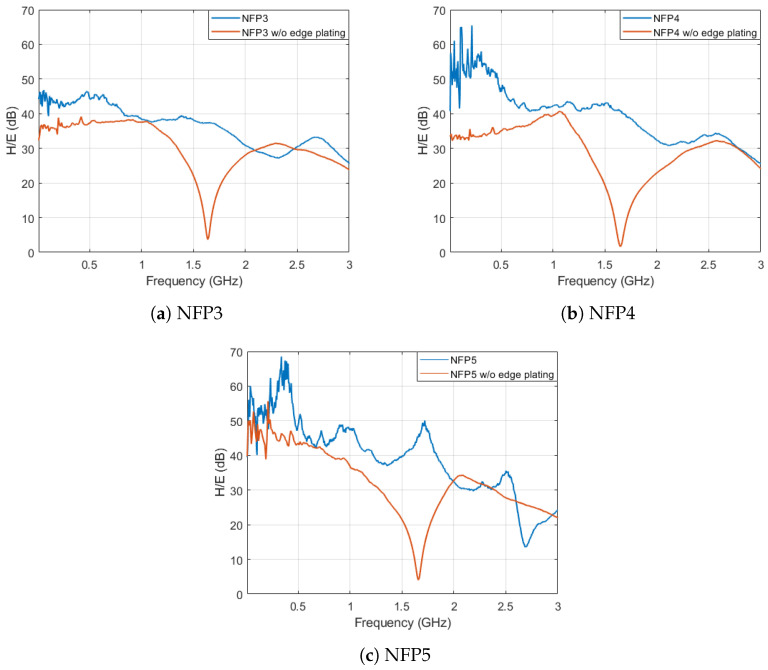
Comparison of probe edge plating influence on the measured electric field suppression ratio of the analyzed probes.

To verify the edge plating influence on the electric field suppression ratio the measured values for probes NFP3-NFP5 are compared. The probes were manufactured with and without edge plating and the measured results are shown in Figure 14. Both the measurement and simulations show that additional edge plating on the probes can considerably increase the electric field suppression ratio of the probe. As mentioned above the edge plating also incorporates a gap at the middle of the loop to decrease the shield currents. The width of the gap on all probes is between 1 and 2 mm due to manufacturing tolerances.

In all 3 cases adding edge plating removed or lowered the resonant behaviour of the probes at 1.65 GHz. The occurring resonance is due to the general probe structure and its LC resonance, as discussed in [3]. The analyzed probes with edge plating have an electric field suppression ratio higher than 40 dB in the frequency range up to 1 GHz and above 30 dB from 1 GHz up to 2 GHz. Above 2 GHz the edge plating shielding effect deteriorates and the results with and without edge plating are comparable. The probe NFP5 shows the average highest electric field suppression ratio up to 2.5 GHz due to the most compact shielding technique. The probes NFP3 and NFP4 have the aforementioned disadvantage of unshielded track and break in the symmetric shield respectively. The measurements contain some noise in the lower frequency band up to 500 MHz due to lower probe sensitivity in this range.

The probe NFP3 electric field suppression is compared to similar probes in [1,13]. The obtained electric field suppression in [13] is at least 15 dB for frequencies below 2.5 GHz and 20 dB for frequencies below 1.2 GHz. Measurement results in [1] show an electric field suppression of more than 30 dB below 6 GHz. The higher values are obtained by incorporating an additional metal can shield on the back of the probe and due to the probe being less sensitive in the frequency range below 3 GHz.

## 5. Conclusions

In this paper a novel design of two-turn shielded near-field probe with high sensitivity and high electric field suppression is presented. Multiple two-turn loop topologies are evaluated in the frequency ranges from 10 MHz to 1 GHz and 3 GHz. The proposed novel two-turn probe design NFP3 has an average sensitivity increase of 10.11 dB compared to the conventional one-turn design in the frequency range from 10 MHz up to 1 GHz. The shielded probes with edge plating show a measured electric field suppression ratio higher than 40 dB from 10 MHz up to 1 GHz. Incorporating edge plating into the probe design removed or lowered the resonance of the electric field response in all cases. The proposed approach with the two-turn probe design allows for an increase in sensitivity, while minimizing the deterioration of the probe spatial resolution. The measurement setup shows a cost-effective PCB probe stand with easy probe and MSL rotation and fixation, which is an alternative to the expensive scanning systems. The proposed probe is compact, cost-effective and can be easily manufactured on a standard four-layer PCB.

It should be noted that the proposed probe, as well as the probes reported in [1,3,9,11,28,29], when used in near field measurements mostly are not exposed to a uniform H-field. Nevertheless, the Equations (8)–(22), based on the assumption of the uniformity of the H-field inside the loop and the invariance of the loop current along the probe length are giving physical insight into the operation principle and measurement error due to the electric field. However, they are not directly applicable in the calculation and prediction of inducted voltages in MSL calibration setup. The calculation of the probe voltage in MSL calibration setups, due to the highly nonuniform H-field and for probe perimeters which are not much smaller than the applied wavelengths, can be based on complicated closed-form expressions [17], or using some established simplified expressions as these proposed in [26] and IEC 61067-6. In this paper, we have run full-wave 3D FEM simulations of the calibration setup, as most other papers dealing with NFP calibration, which takes into account previously mentioned effects. Our future work will include further development of calibration methods and setups using MSL-s and TEM cells.

## Figures and Tables

**Table 1:** Dimensions of the conventional probe.

Parameter	Dimension (mm)	Parameter	Dimension (mm)
$w_{p}$	15.2	$w_{c}$	0.3
$l_{p}$	70	$l_{c}$	1
$w_{L}$	3	$w_{t}$	0.3
$l_{L}$	3	$l_{t}$	63
$w_{g}$	2.1	$h_{d_{1}}$	0.5
$l_{g}$	2.1	$h_{d_{2}}$	0.5

**Table 2:** Optimization variables and results for the conventional probe.

Optimization Variable	Range (mm)	Results (mm)
$w_{p}$	14–16	15.2
$l_{p}$	65–75	70
$w_{t}$	0.2–0.5	0.3
$w_{g}$	1.7–2.1	2.1

**Table 3:** Conventional PCB probe layer stack-up.

Parameter	Layer Name	Material	Thickness (mm)
$h_{ts}$	Top Solder	Solder Resist	0.01
$h_{L1}$	L1-Top Layer	Copper	0.018
$h_{d1}$	Dielectric 1	FR4	0.5
$h_{L2}$	L2-Mid Layer 1	Copper	0.018
$h_{d2}$	Dielectric 2	FR4	0.5
$h_{L3}$	L3-Mid Layer 2	Copper	0.018
$h_{d3}$	Dielectric 3	FR4	0.5
$h_{L4}$	L4-Bottom Layer 1	Copper	0.018
$h_{bs}$	Bottom Solder	Solder Resist	0.01

**Table 4:** Sensitivity of the designed probes at key frequencies obtained from both simulation and measurement results.

		Frequency		
Probe	0.5 GHz	1 GHz	2 GHz	3 GHz
NFP1—simulation	−40.8	−33.9	−30.5	−31.1
NFP1—measurement	−40.4	−32.8	−31.5	−30.2
NFP2—simulation	−32.7	−28.2	−25.3	−39.6
NFP2—measurement	−33.6	−28.7	−23.8	−25.2
NFP3—simulation	−29.5	−26.5	−27.5	−31.6
NFP3—measurement	−29.1	−25.7	−29.2	−30.1
NFP4—simulation	−32.1	−29.0	−29.1	−33.7
NFP4—measurement	−32.6	−28.1	−31.1	−31.8
NFP5—simulation	−33.3	−30.4	−35.0	−45.3
NFP5—measurement	−33.2	−29.1	−36.6	−39.3

## Data Availability

The data presented in this study are available on request from the corresponding author.
